# Diagnostic accuracy of ^18^F–FDG PET/CT and MR imaging in patients with adenoid cystic carcinoma

**DOI:** 10.1186/s12885-017-3890-4

**Published:** 2017-12-22

**Authors:** Verena Ruhlmann, Thorsten D. Poeppel, Johannes Veit, James Nagarajah, Lale Umutlu, Thomas K. Hoffmann, Andreas Bockisch, Ken Herrmann, Wolfgang Sauerwein

**Affiliations:** 1 Department of Nuclear Medicine, University Duisburg-Essen, Medical Faculty, University Hospital Essen, Hufelandstrasse 55, 45147 Essen, Germany; 2 Department of Oto-Rhino-Laryngology, Head and Neck Surgery, University Hospital Ulm, Frauensteige 12, 89070 Ulm, Germany; 3 Department of Nuclear Medicine, Radboud University Nijmegen Medical Centre, Geert Grooteplein 8, 6525 GA Nijmegen, the Netherlands; 4 Department of Diagnostic and Interventional Radiology and Neuroradiology, University Duisburg-Essen, University Hospital Essen, Hufelandstrasse 55, 45147 Essen, Germany; 5 Department of Radiation Oncology, University Duisburg-Essen, University Hospital Essen, Hufelandstrasse 55, 45147 Essen, Germany

**Keywords:** PET/CT, MRI, Adenoid cystic carcinoma

## Abstract

**Background:**

The aim of this study was to evaluate the value of 18F–FDG PET/CT (PET/CT) and MRI for local and/or whole-body restaging of adenoid cystic carcinoma of the head and neck (ACC).

**Methods:**

Thirty-six patients with ACC underwent conventional MRI of the head and neck and a whole-body PET/CT and were analysed with regards to detection of a local tumor recurrence, lymph node or distant metastases. A consensus interpretation of all available imaging data was used as reference standard. Sensitivity, specificity, diagnostic accuracy, positive and negative predictive values were calculated for MRI and PET/CT.

**Results:**

The sensitivity of PET/CT and MRI was 96% (89%), specificity 89% (89%), PPV 96% (96%), NPV 89% (73%) and accuracy 94% (89%) for detection of local tumors. Additionally, PET/CT revealed lymph node metastases in one patient and distant metastases in 9/36 patients. In three patients secondary primaries were found.

**Conclusions:**

Whole-body PET/CT in addition to MRI of the head and neck improves detection of local tumour and metastastic spread in ACC.

## Background

Adenoid cystic carcinoma (ACC) is a rare type of cancer mainly located within secretory glands, most commonly the minor but also the major salivary glands of the head and neck [1]. Each year, about 1200 people are diagnosed with ACC in the United States. ACC is a (generally) well-differentiated and slowly growing tumour with a relatively indolent but relentless course. The tumour is characterised by a marked tendency for perineural invasion with the risk of incomplete resection and (late) local and distant recurrences [2–4].

Due to the tendency of this tumour to spread along nerve tracts, the complete radical resection of the primary tumour as the main therapeutic approach is very challenging and results in frequent local recurrences. Adjuvant radiotherapy is commonly applied after surgery and leads to improved local control. Furthermore, in some cases, surgery is not feasible to tumour location, therefore primary radiation (e.g. using high linear energy transfer (LET) beams (fast neutrons or carbon ions) [5–7] is the only treatment option. Chemotherapy has only limited effects in trials so far [8–10]. Sub-cellular and genetic characteristics of the ACC can be used for targeted systemic therapy efforts and have in part already been considered in clinical trials [11]. Hence, so far no breakthrough has been achieved in systemic therapy for this entity.

In contrast to 10- to 20-year survival rates (about 40% at 15 years), 5-year survival rates in patients with ACC are high with around 89% [12, 13], reflecting a prolonged course of metastatic disease and late local recurrences. Another specific feature of ACC is the relatively rare occurrence of regional lymph nodes metastases although micro metastases might be detected in a relevant number of patients if neck dissection is performed [14]. Even after successful local therapy distant metastases often occur metachronously in lung and sometimes in the liver and limit long-term prognosis. According to the existing literature, distant metastases in ACC are rare at initial diagnosis and depend on disease duration and primary site, although some authors state that current diagnostic means might not be sensitive enough to detect them [15, 16].

Due to the diffuse growth pattern the detection of local recurrence, especially differentiation between scar formation, radiation effects and vital tumour is limited and routine imaging techniques (computed tomography (CT), magnetic resonance imaging (MRI) and ultrasound (US)) suffer from low specificity [17–19]. Thus, in case of suspected recurrence it is anticipated that metabolic information provided by ^18^F–fluorodeoxyglucose (FDG) positron emission tomography (PET) could improve the distinction between benign and malignant lesions [17, 20, 21]. Published reports on FDG uptake in ACC are rare [22–25], and many of these are only case series with a small number of patients. The existing literature hints that most squamous cell carcinomas are intensely FDG avid, whereas adenoid cystic carcinoma and mucoepidermoid carcinoma show variable uptake depending on the grade of differentiation [26]. In a recently published study by Kim et al. [27] the pretreatment SUVmax of FDG-PET was as a predictor of distant metastasis in ACC of the head and neck.

In this study we evaluate the diagnostic accuracy of FDG PET/CT and MRI for restaging ACC patients in a relatively large population with particular focus on local and whole-body staging.

## Materials and methods

### Patient population

Imaging data of 36 consecutive patients (19 females, 17 males, mean age 57 ± 12 years) suffering from ACC of the head and neck (histologically confirmed initial localisations: salivary glands (parotid and submandibular gland; *n* = 18), small salivary glands: oropharynx (*n* = 7), trachea (*n* = 2), paranasal sinus (*n* = 4), eye socket (n = 2) and upper jaw (*n* = 3)) were retrospectively analysed. The diagnostic imaging procedures were performed within the context of routine clinical procedures within a short time interval in the case of restaging of ACC when suspecting recurrences (*n* = 16) or approximately two months after local tumour resection prior to adjuvant radiation therapy (*n* = 20). Therefore, ethics approval was waived.

### MRI studies

All patients underwent a conventional MRI of the head and neck. MRI was performed in a whole-body 1.5 Tesla MR scanner using the following sequence protocol:

1. A T1-weighted Spin Echo (T1 SE) in coronal slice orientation (time of repetition (TR) 425 ms, echo time TE) 15 ms, slice thickness 5 mm, matrix size 512, field of view (FOV) 280).
2. An axial T1-weighted SE (TR 455 ms, TE 11 ms, slice thickness 5 mm, matrix size 512, FOV 230).
3. An axial T2-weighted SE (TR 3900 ms, TE 87 ms, slice thickness 5 mm, matrix size 512, FOV 260).
4. An axial post contrast T1-weighted SE with fat saturation (TR 474 ms, TE 11 ms, slice thickness 5 mm, matrix size 512, FOV 230) after i.v. administration of 0.1 mmol/kg body weight of gadobutrol (Gadovist, Bayer Healthcare, Leverkusen, Germany).
5. A coronal post-contrast T1-weighted SE TR (TR 710 ms, TE 15 ms, slice thickness 5 mm, matrix size 512, FOV 280).

### PET/CT studies

Dedicated head and neck and whole-body FDG PET/CT scans were performed using a Biograph mCT™ (Siemens AG, Healthcare Sector, Erlangen, Germany). The head and neck scan was performed 60 ± 5 min after tracer injection (weight-dependent FDG dose, mean activity 290 ± 45 MBq) and 40 s after intravenous contrast agent injection (60 ml Ultravist®; Bayer Healthcare Deutschland, Leverkusen, Germany) from the skull base to the aortic arch (slice thickness 3 mm). To minimize artefacts, the patient’s arms were placed beside the body. Afterwards, the patients were encouraged to place the arms over their head for the following whole-body examination. The scan ranged from the upper thorax to the thighs and was performed 70 s after injection of an additional dose of 70 ml contrast agent (slice thickness 5 mm). In both protocols, the manufacturer-supplied dose reduction techniques CareDose 4D™and CareKV™ were used (presets 210 mAs, 120 kV; Siemens AG). Blood glucose was below 150 mg/dl at the time of tracer injection. PET data were acquired for 4 min per bed position in the head and neck area and for 2 min per bed position in the rest of the body. For reconstruction, attenuation weighted ordered-subsets expectation maximization (AW-OSEM) iterative algorithm with 4 iterations and 8 subsets, Gaussian filter with 4.0 mm full width at half maximum (FWHM) and scatter correction were used.

Immediately after the PET scan a thoracic full-dose CT scan was performed in the forced inspiratory position with the following parameters: 120 kV, automatic mA/s adjustment (Care Dose 4D™, preset: 70 mAs), 5-mm slice thickness; reconstruction in lung window.

### Image analysis

Board certified physicians in nuclear medicine and radiology visually interpreted the PET/CT and MRI data, being blinded to the results of the other imaging examinations. The presence of residual/recurrent tumours, number of regional lymph node metastases and distant metastases were counted separately in both CT and MRI.

On MR images, a lesion in a suspected site of tumour with a hypo- to isotense signal in the T1-weighted image, a slightly to markedly hyperintense signal in the T2-weighted image and an enhancement in the contrast-enhanced T1-weighted image was considered as malignant. On CT and MR images, increased short-axis diameter (>10 mm), central necrosis, irregular shape and the lack of a fatty hilus sign and increased contrast agent uptake were considered as signs of malignancy in lymph nodes.

On the hybrid images (PET/CT), central necrosis, focal soft tissue increase and focal FDG-uptake were considered as indicators of malignancy in local cancer recurrence. In a PET-specific semi-quantitative approach, the maximum standardized uptake values (SUVmax) of all tumour lesions/suspected sites of metastasis or focally increased tracer uptake visually defined above the mediastinal blood pool were determined by drawing spherical volumes of interest (VOI) that closely encircled a lesion.

### Reference standard

A consensus interpretation of all available imaging data (prior examinations and follow-up examinations (median 12, range 3–105 months): MRI of the head and neck, thoracic CT, whole-body-PET/CT, ultrasound) adopted by all readers was used as reference standard.

### Statistical analysis

Sensitivity, specificity, positive predictive value (PPV), negative predictive value (NPV) and diagnostic accuracy were calculated for MRI, PET, CT and PET/CT using Microsoft Excel (15.29.1).

## Results

### Recurrent or residual tumour

**Fig. 1:**
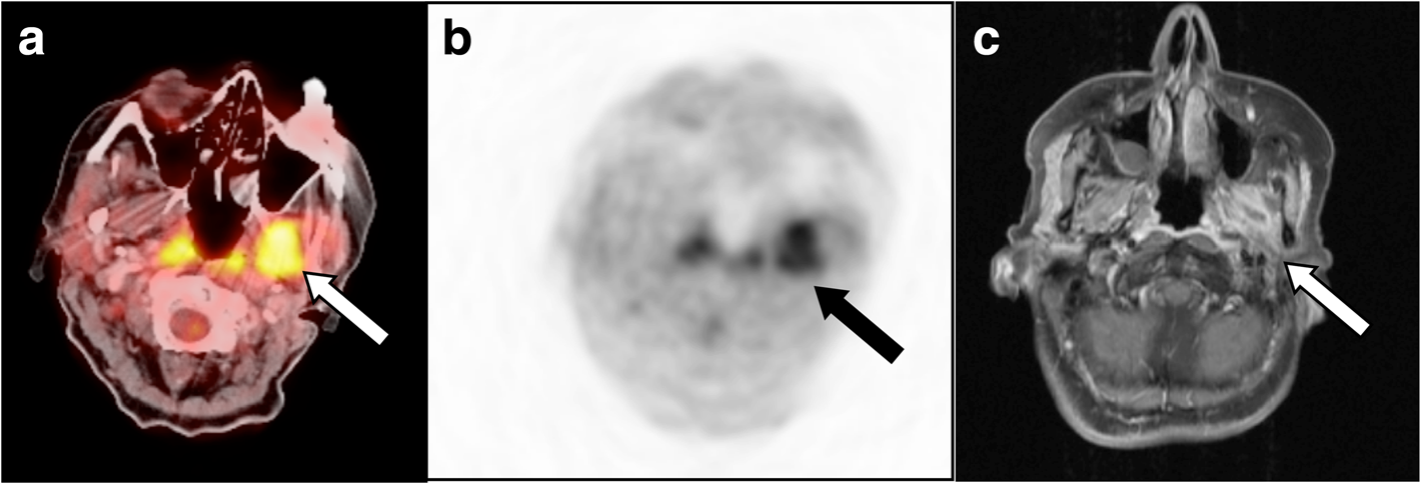
ACC recurrence: PET/CT imaging was true-positive, indistinctive finding on MRI. Patient after resection of an adenoid cystic carcinoma in the left parotid gland and after combined neutron/proton therapy. Recurrence is seen on the fused PET/CT (**a**) and PET image (**b**) with a focally increased FDG-uptake (arrow), but not on the contrast-enhanced T1-weighted MR image (**c**). In MRI, the finding was evaluated as radiation necrosis

**Fig. 2:**
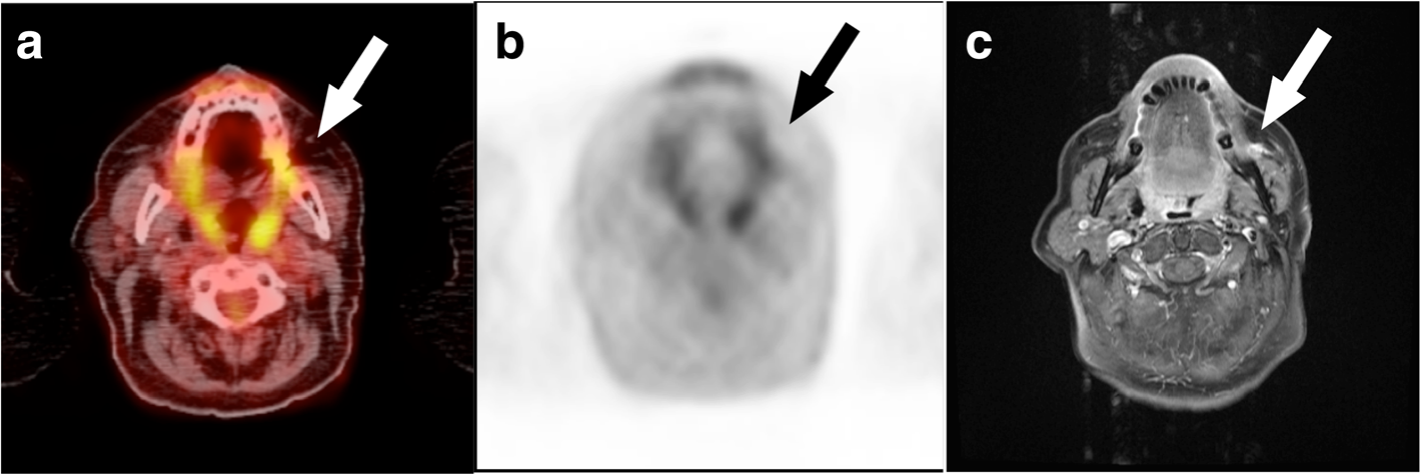
No tumour recurrence: PET/CT imaging was true-negative, indistinctive finding on MRI. Patient after multiple resections of recurrent tumours of a left buccal adenoid cystic carcinoma. A hyperintense lesion is seen on the contrast-enhanced T1-weighted MR image (**c**) (arrow), but there was no pathological finding on the fused PET/CT (**a**) and PET image (**b**). The follow-up confirmed no recurrence

A recurrent or residual tumour was present in 27 of the 36 patients according to the reference standard. MRI detected 24 of 27 tumour lesions. In the remaining three cases with tumour recurrence the MRI findings were indistinct (an illustrative example is given in Fig. 1). MRI was negative in 8 of 9 the patients without signs of tumour and indistinct in one patient (Fig. 2). Thus, the sensitivity was 89%, specificity 89%, PPV 96%, NPV 73% and accuracy 89%.

**Fig. 3:**
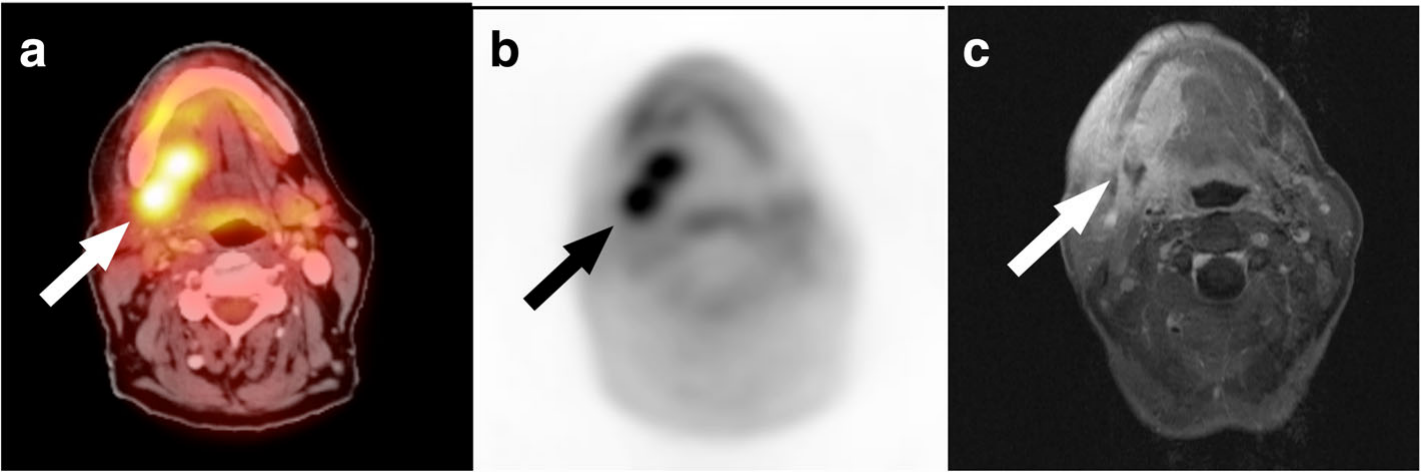
ACC tumour remaining: PET/CT and MR imaging was true-positive. Patient after resection of an adenoid cystic carcinoma, now with a tumour remnant on the right root of the tongue prior to neutron therapy. The tumour mass is seen on the fused PET/CT (**a**) and PET image (**b**) with a focally increased FDG-uptake (SUVmax 14.1) and contrast-enhanced T1-weighted MR image (**c**) (arrow)

**Fig. 4:**
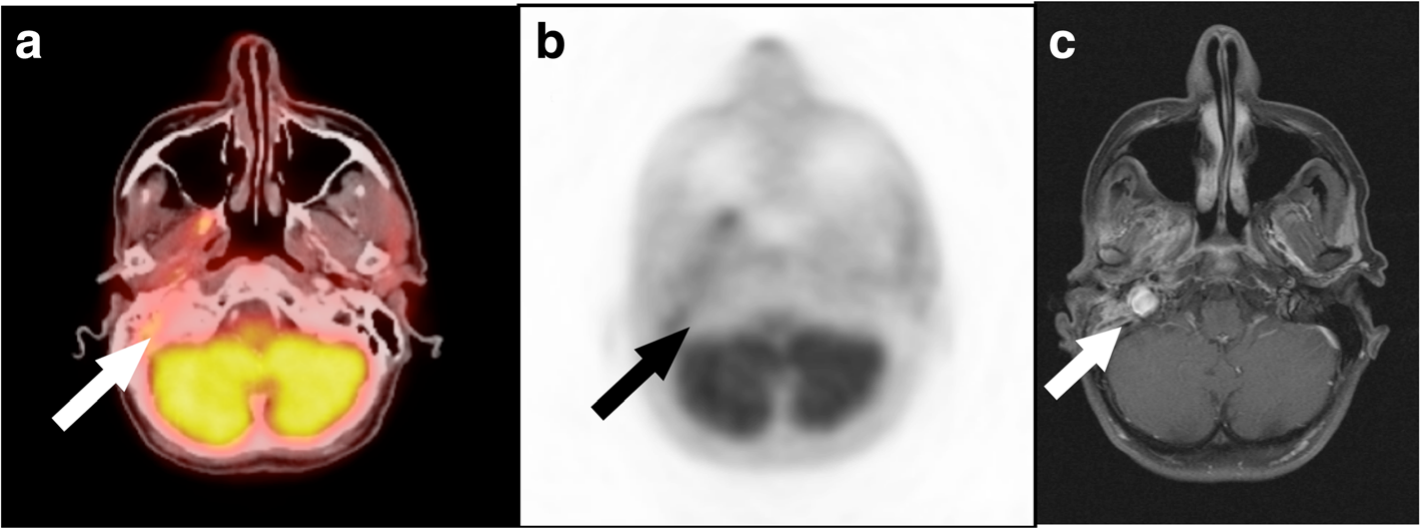
ACC recurrence: PET/CT imaging was false-negative and MRI true-positive. Patient after multiple resections of the primary and recurrent tumours of an ACC in the right parotid gland area. A false-negative finding is seen on the on the fused PET/CT (**a**) and PET image (**b**), but there was a true-positive finding on the contrast-enhanced T1-weighted MR image (**c**) with a hyperintense tumour recurrence (arrow)

**Fig. 5:**
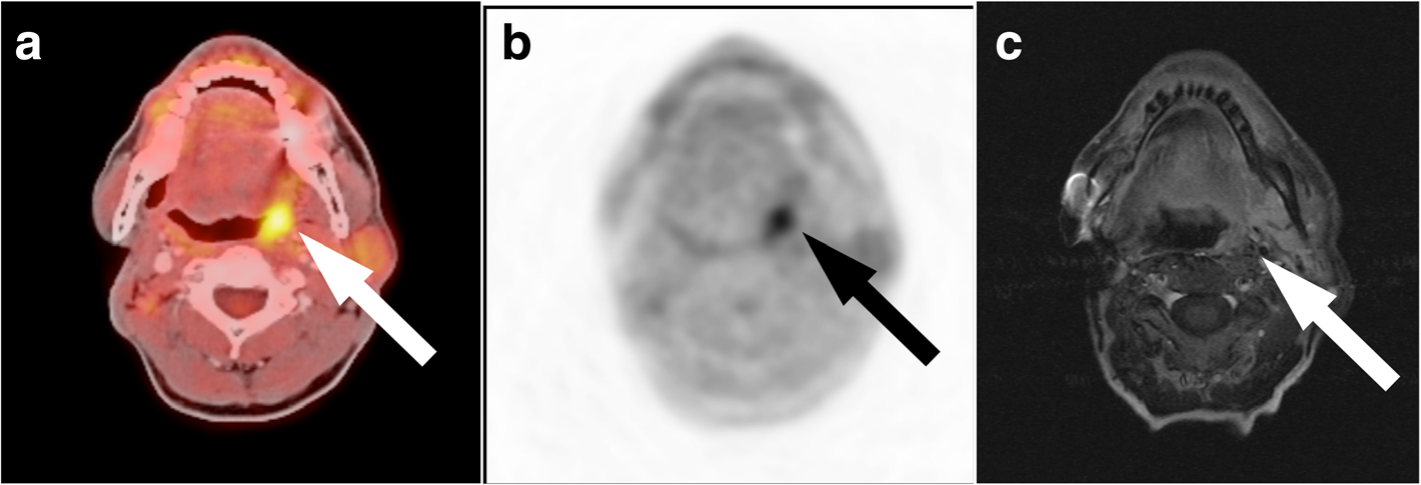
No tumour recurrence: PET/CT imaging was false-positive and MRI true-negative. Patient after resection of an ACC in the right parotid gland and mandibula. A contralateral false-positive finding is seen on the PET (**b**) and fused PET/CT image (**a**) with a focally increased FDG-uptake (SUVmax 5.4; arrow), but there was no pathological finding on the contrast-enhanced T1-weighted MR image (**c**)

PET/CT detected 26 of 27 tumour lesions with increased FDG uptake (Fig. 3). One patient did not show any pathologically increased tracer uptake in the tumour lesion (in the former parotid gland area with penetration of the base of the skull, the mastoid and neurocranium) that was only detected on MRI (Fig. 4). Mean SUVmax across all lesions was 6.8 ± 3.4 (median 5.7, range 2.0–15.0). PET/CT was negative in 8 of 9 patients without a tumour. PET/CT was false-positive in one patient (Fig. 5). Thus, the corresponding sensitivity was 96%, specificity 89% and accuracy 94%. PPV and NPV calculated to 96% and 89%, respectively.

### Lymph node metastases

In one patient, regional cervical lymph node metastases were dectected in accordance with the reference standard. On the PET component this patient with the primary tumour in the right parotid gland presented increased FDG uptake (SUVmax 6.0), whereas the the lymph node was according to CT and MRI size criteria not suspicious.

### Distant metastases

PET/CT detected distant metastases in 9 out of 36 patients with manifestations in the lung (*n* = 8), bone (*n* = 5), adrenal gland (*n* = 1), kidney (n = 1), and liver (*n* = 1), as well as distant lymph node metastases (*n* = 1) in the mediastinum (primary localisation in the submandibular gland). Corresponding mean SUVmax was 3.9 ± 2.3 (median 3.8, range 1.2–9.9). Four patients presented lung metastases that were not known at time of first diagnosis but discriminated by PET/CT after local tumour resection prior to adjuvant radiation therapy.

In all patients with distant metastases, the lesions could be defined as malignant on combined PET/CT. In case of lung metastases the CT was indistinctive in 2 patients (differential diagnosis: granuloma), but the PET component helped the lesion characterization as malignant. In two cases with disseminated lung metastases, the smallest malignant lesions could only be detected on CT.

All patients with distant metastases had no synchronical regional lymph node metastases, and 4/9 patients had no local recurrence.

In addition to the main diagnosis a secondary primary carcinoma was found in three patients. In these cases with histopathologically confirmed cervical carcinoma, prostate carcinoma and breast cancer preferential treatment was conducted.

## Discussion

This study demonstrates the high sensitivity of whole-body FDG PET/CT in detecting both recurrent/residual tumours and regional metastatic spread in patients with ACC. FDG PET/CT also outperformed head neck MRI for local staging and restaging. Moreover, the whole body imaging approach enables the detection of distant metastases even at an early stage and in the follow up.

Local tumours were present in 75% of the patients in our study population. Of these, 63% had residual tumours, and 37% had tumour recurrence. The detection rate of a residual or recurrent tumour on PET/CT was highly accurate with only one false-negative and one false-positive finding. PET/CT was only false-negative in one patient with tumour recurrence in the head and neck area. PET/CT was false-positive in only one case in the head and neck area, possibly due to a physiological hyperfunctional pharyngeal reaction and perfusion after contralateral resection of the primary tumour. MRI had four indistinct findings in the former local tumour region. The diagnostic performance of PET/CT was comparable to MRI in local restaging with a higher sensitivity (96% v. 89%) and diagnostic accuracy (94% vs. 89%) due to less indistinct findings.

Locoregional lymph node metastases were present in only one patient in our study population with a true-positive finding on PET and false-negative finding on CT or MRI due to size criteria. This low incidence of locoregional lymph node metastases is in line with previous reports that described that an ACC rarely metastasises to regional lymph nodes [28, 29]. The ability of PET to detect metastases in lymph nodes that are inconspicuous in morphologic imaging has been shown in a variety of cancers [17, 30, 31].

Combined whole-body PET/CT detected all distant metastases in our study. There were only two cases with disseminated metastases of the lung in which not all of the CT-detectable lesions were also FDG positive due to the limited spatial resolution. In two patients the lung metastases showed a pathologically increased FDG uptake, whereas the CT was indistinctive (differential diagnosis: granuloma). Distant metastases were present in 25% of the patients in our study population. Surprisingly, in the patient group with the primary examination after local tumour resection prior to radiation therapy, 11% of the patients already showed distant metastases in the lung. In three patients secondary carcinomas were found with PET/CT allowing early therapeutic interventions. Previous reports have described overall rate of distant metastases in 25 to 50% of patients with ACC [29], but the occurrence mainly depends on the follow-up period. In fact, in 35 to 50% of patients with ACC, distant metastases were usually found within a follow-up period of more than 15 to 20 years [32]. These studies, which were published several years ago, used conventional diagnostics such as X-ray with comparably low specificity and sensitivity for staging the lung of ACC patients. Therefore, slow-growing metastases might have been misinterpreted as newly occurred metastases at a late time point, although other diagnostic imaging methods like CT might have shown a metastatic disease at an earlier time point in their clinical course or even at time of initial diagnosis.

According to Spiro et al. [29], large primary tumour size and locoregional treatment failure are the most important predisposing factors for outcome. However, distant metastases may develop despite successful locoregional tumour therapy. As in our study, the lungs are the most commonly reported sites to harbour distant metastases. These patients may remain asymptomatic for a long time during their clinical course. However, the onset of symptoms or the development of other visceral metastases usually results in a short survival period. Thus, whole body imaging seems advisable in restaging and follow-up of ACC patients to better understand the course of disease and find optimal therapy strategies, as was recently highlighted in a report from Tewari et al. [33].

In our study, almost all lesions were FDG-avid, and the majority showed a moderately to high FDG uptake. Previously published literature predominantly case reports or case series on FDG uptake in this advancing but relentlessly growing tumour with a propensity for perineural invasion comprise only very few patients [22–25]. In larger studies ACC often represent only a minority within a group of mixed head and neck cancers [25]. The existing literature of experience with PET imaging show that adenoid cystic carcinoma and mucoepidermoid carcinoma showed variable FDG-uptake depending on the grade of differentiation [26] in comparison to most squamous cell carcinomas that showed high uptake. Due to the complex development of the disease in patients with ACC an appropiate diagnostic imaging is required. The evaluation of changes in tissues and structures in the tumour environment, especially after extended tumour resection in the head and neck area, surgical procedures with transplants or external beam radiotherapy, is a big challenge since US, conventional CT and MRI provide mainly just morphological information and exactly here PET imaging can improve the diagnostic work-up as a tool that depict metabolism information.

A limitation of this study is the nevertheless limited number of patients due to the rarity of this malignant disease. This study cohort comprised 36 patients which is more than any other study. The small sample size did not permit an analysis of the FDG uptake depending on the histopathological ACC subtypes (cribriform, solid and tubular). Another limitation is the reference standard lacking histopathological confirmation. However, the consensus interpretation (adopted by all readers) based on profound imaging data including prior and follow-up examinations (median 12, range 3–105 months).

Interestingly, FDG PET/CT depicted all lesions visualized by the standard imaging tools head and neck is MRI and if applicable thoracic CT. This underlines the potential of whole-body FDG-PET/CT improving the diagnostic accuracy of restaging ACC. Whereas MRI is commonly needed preoperatively for the assessment of tumour delineation and relevant tumour invasion of surrounding structures it is not a routine whole-body imaging technique. The advantages of FDG-PET/CT are the examination of the whole body at one time and at a high sensitivity. Furthermore, our study demonstrates that due to certain weaknesses the conventional CT and MR imaging of the head and neck could benefit from the additional metabolism information provided by the PET component. Thus, in the time of installation of integrated whole-body PET/MR systems, it would be of interest to determine whether a single whole-body PET/MRI could replace a whole-body PET/CT by combining the advantages of both modalities.

## Conclusions

Our study demonstrates the potential of FDG-PET/CT as important diagnostic tool in the restaging of ACC patients. FDG PET/CT has a higher sensitivity and diagnostic accuracy than MRI for the loco-regional tumour staging and provides valuable additional information as a whole-body technique.
